# One step forward for nanopore protein sequencing

**DOI:** 10.1002/ctm2.1615

**Published:** 2024-03-11

**Authors:** Ziyi Li, Yakun Yi, Lei Liu, Hai‐Chen Wu

**Affiliations:** ^1^ Beijing National Laboratory for Molecular Sciences Key Laboratory of Analytical Chemistry for Living Biosystems Institute of Chemistry Chinese Academy of Sciences Beijing China; ^2^ University of Chinese Academy of Sciences Beijing China; ^3^ Key Laboratory for Biomedical Effects of Nanomaterials and Nanosafety Institute of High Energy Physics Chinese Academy of Sciences Beijing China

**Keywords:** nanopore technology, protein sequencing

1

With the successful completion of the Human Genome Project, decoding entire human genomes has become a reality. According to the central dogma of molecular biology, genetic information flows from nucleic acid sequences to proteins, allowing for the decoding of the amino acid composition sequence from gene sequences. However, additional information remains concealed in subsequent processes such as post‐translational modifications, protein splicing, and degradation. This hidden information necessitates exploration through high‐throughput and high‐sensitivity proteomics research.

Since the inception of the first protein sequencing method, Edman Degradation,^1^ in 1949, researchers have devoted considerable efforts to decode proteins. While mass spectrometry remains the gold standard in protein sequencing, it faces limitations in dynamic range, sequencing length, and accuracy.^2^ Recent years have witnessed the emergence of new methods, including fluorosequencing,^3^ single‐molecule peptide fingerprinting,^4^ tunnelling current^5^ and nanopore‐based protein sequencing.^6^, ^7^, ^8^, ^9^, ^10^, ^11^

While the success of nanopore DNA sequencing has driven the exploration of nanopore technology for protein sequencing, the latter presents unique challenges. Protein sequencing introduces additional complexity to nanopore systems when compared to DNA sequencing. This complexity stems not only from the existence of 20 distinctive amino acids, in contrast to the four nucleic acids in DNA, resulting in an exponential growth in data volume. Moreover, peptides in proteins exhibit heterogeneous charges, unlike the uniform negative charges of DNA molecules, thereby presenting challenges for directional translocation in nanopore systems.

Inspired by DNA sequencing, nanopore protein sequencing has evolved, broadly categorized into two distinct methodologies: full‐length chain sequencing and enzyme‐assisted sequencing (Figure 1). The full‐length chain sequencing strategy involves directing the unfolded protein chain through the nanopore. Three groups independently employed a DNA motor to ratchet a DNA‐peptide conjugate through a nanopore, allowing for the reading of the peptide sequence.^6^, ^10^, ^11^ Other attempts include the use of electroosmotic force achieved by adding guanidinium chloride to the buffer solution^8^ or by mutating protein pores.^12^ On the other hand, enzyme‐assisted sequencing entails enzymatic cleavage of proteins into individual amino acids, subsequently identifying them sequentially. In a recent study, Huang et al. engineered the MspA nanopore with nitrilotriacetic acid, later combining it with Ni^2+^, which demonstrated the ability to distinguish all 20 proteinogenic amino acids.^9^ While these advancements have pushed the boundaries of nanopore protein sequencing, none of them have been able to reveal sequence information. Recently, we proposed an alternative strategy for peptide sequencing based on a combination of enzymatic cleavage and host–guest interaction‐assisted nanopore sensing, aiming to achieve comprehensive peptide sequencing.^7^

**FIGURE 1:**
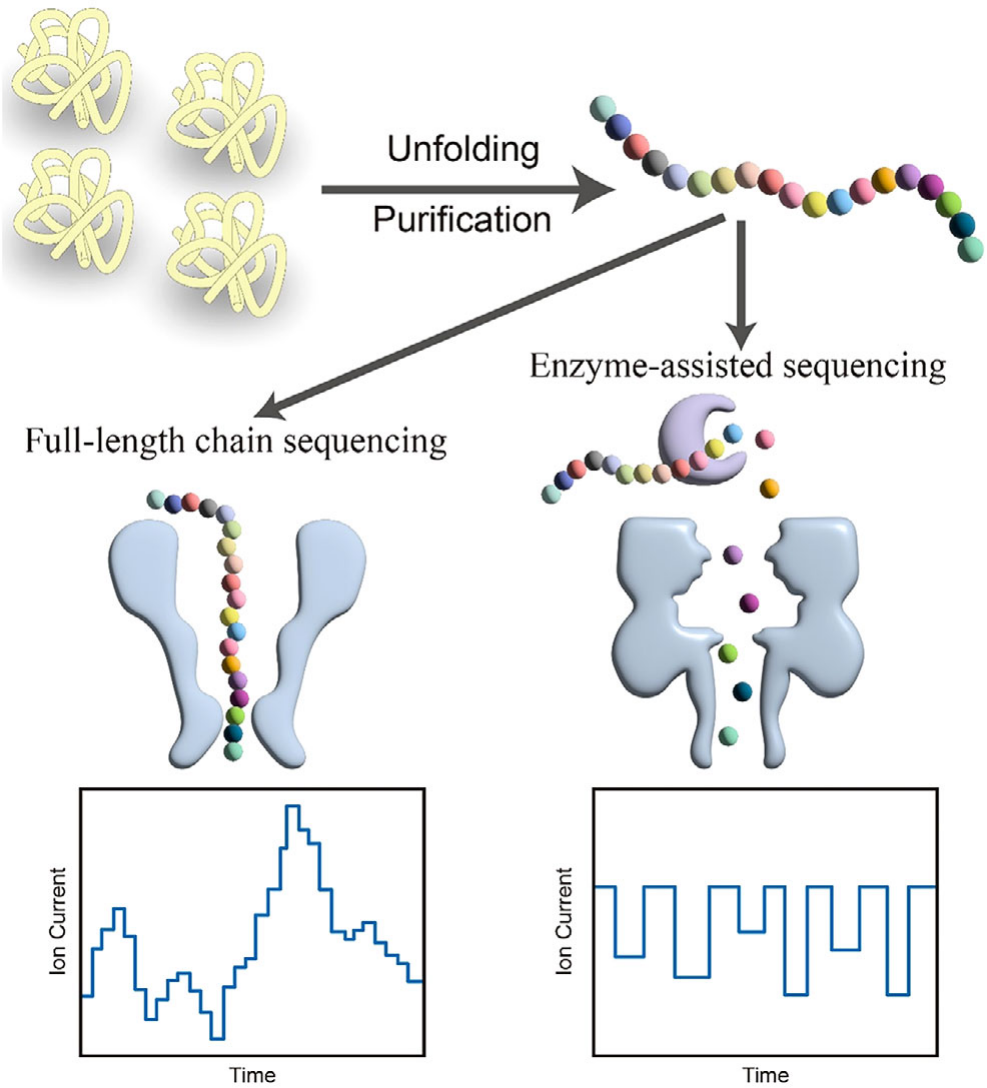
Strategies in nanopore protein sequencing. The protein was first unfolded and purified to obtain individual peptides. Subsequently, these peptides were passed through a nanopore to generate a full‐length read, resulting in a series of current signals that encapsulate sequencing information (left). In another method, proteases are employed to sequentially digest the peptides into amino acids. These individual amino acids then pass through the nanopore, generating characteristic signals (right).

In our previous research, we found that the host‐guest interaction between phenylalanine (F) and cucurbit[7]uril (CB[7]) significantly improves nanopore recognition, resulting in consistent and prolonged current events.^13^ Through meticulous experimentation, the FGXD_8_ model peptide demonstrated optimal performance for discriminating X, where ‘X’ represents any of the 20 proteinogenic amino acids (Figure 2A). The negative‐charged polyaspartic acid chain drove translocation in an electric field, while strong interactions between FG and CB[7] stabilized the peptide⊂CB[7] complex at the wildtype α‐hemolysin (WT αHL) constriction, providing the best resolution. Using this probe with the αHL and a specific mutant (M113F)_7_ allowed for the discrimination of all 20 proteinogenic amino acids in the FGXD_8_ sequence (Figure 2B).

**FIGURE 2:**
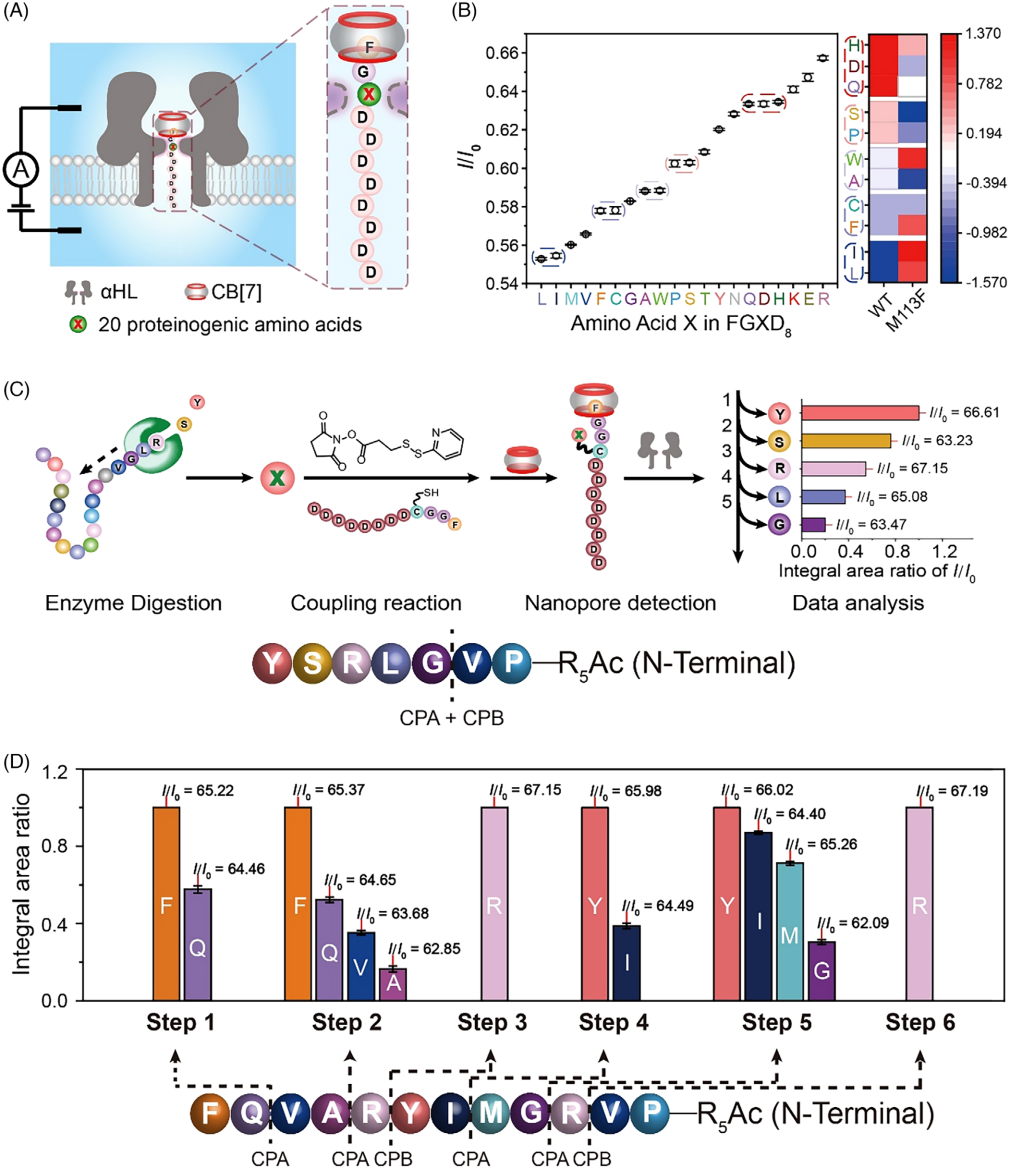
Peptide sequencing based on a combination of enzymatic cleavage and host–guest interaction‐assisted nanopore sensing. (A) Schematic of the experimental setup and peptide constructs used to discriminate the 20 amino acids. (B) Experimentally determined mean *I*/*I*_0_ values and their s.d. in an ascending order generated by the translocation of FGXD_8_⊂CB[7] through wildtype α‐hemolysin (WT αHL) (left). The heat map of mean *I*/*I*_0_ produced by FGXD_8_⊂CB[7] through WT and (M113F)_7_ αHL (right). (C) Schematic illustration of the process for peptide sequencing using the probe peptide FGGCD_8_ and the mixed enzymes digestion strategy. The model peptide was digested with a mixture of carboxypeptidase A (CPA) and CPB. (D) Stepwise enzymatic digestion strategy. The peptide was digested by either dilute CPA or CPB during each step. The sequencing was completed in six steps, with each step revealing the identities of one or two amino acids.

Motivated by these outcomes, we adapted this methodology for detecting free amino acids. Conjugating the amino group of free amino acids with the sulfhydryl group on FGGCD_8_ created the FGGC(X)D_8_ probe, efficiently attaching nearly all proteinogenic amino acids (Figure 2C). With nanopore mutants and altered experimental conditions, we achieved distinct and reliable differentiation of the 20 proteinogenic amino acids.

Identifying free amino acids is a preliminary step for protein sequencing. The challenge lies in obtaining sequential amino acid information. Expecting sequential peptide digestion by protease, we aimed to identify digested amino acids using the FGGC(X)D_8_ probe. When employing carboxypeptidase A and B (CPA and CPB) to cleave peptides from the C‐terminus, we faced a significant challenge: excessively rapid enzymatic digestion hindered isolating single amino acids. Fortunately, experiments revealed a correlation between amino acid abundance and position within the peptide chain (Figure 2C). Despite rapid cleavage, position‐dependent abundance variability suggested a pathway for deducing peptide sequences based on relative amino acid quantities. The inclusion of D_8_ in our probe, known for strong negative charges, plays a vital role in averaging charges across the 20 natural amino acids and enhancing sequencing efficiency.

While effective in accurately sequencing short peptides, our strategy faced dephasing beyond 8–10 digested amino acids. To address this, we devised a stepwise enzyme digestion approach (Figure 2D). Exploiting distinct properties of CPA and CPB, we introduced CPA to determine the sequence preceding arginine (R), followed by CPB to specifically cut the R residue. Repeating these cycles significantly extended our capability to accurately sequence much longer peptides.

In conclusion, our innovative peptide sequencing strategies mark a promising frontier in proteomics. Future optimization includes an integrated nanopore chip for high throughput, artificially modified enzymes to control the digestion speed, and improved probe design for reduced sequencing time. Integration of advanced data analysis algorithms and machine learning is crucial for handling the exponential growth in data volume from complex protein sequencing. Beyond basic research, in the clinical realm, nanopore protein sequencing has the potential to revolutionize diagnostics, enabling rapid and precise identification of disease biomarkers for earlier detection and personalized treatment plans, leading to improved patient outcomes. Despite the challenges, nanopore protein sequencing shows a bright future, promising to unlock the full narrative encoded within proteins and providing unprecedented insights into the molecular machinery of life, ushering in a new era of biomedical breakthroughs.

## AUTHOR CONTRIBUTIONS

Ziyi Li, Yakun Yi and Hai‐Chen Wu conceived the manuscript and composed the figures. Ziyi Li, Lei Liu and Hai‐Chen Wu wrote the manuscript and approved the final draft.

## CONFLICT OF INTEREST STATEMENT

Hai‐Chen Wu, Yakun Yi and Ziyi Li have filed patents describing the strategy for the nanopore‐based peptide sequencing. Lei Liu declares no conflict of interest.

## ETHICS STATEMENT

This article does not contain any research involving humans or animals.
